# Multi-criteria discovery, design and manufacturing to realise nanomaterial potential

**DOI:** 10.1038/s44172-023-00128-6

**Published:** 2023-11-06

**Authors:** Robert Pilling, Stuart R. Coles, Marc R. Knecht, Siddharth V. Patwardhan

**Affiliations:** 1 Department of Chemical and Biological Engineering, University of Sheffield, Sheffield, S1 3JD UK; 2 WMG, International Manufacturing Centre, University of Warwick, Coventry, CV4 7AL UK; 3 Department of Chemistry, University of Miami, 1301 Memorial Drive, Coral Gables, FL 33146 USA

**Keywords:** Chemical engineering, Nanoscale materials

## Abstract

Nanomaterial solutions to sustainable development goals are hindered in their path to commercialisation by an early-stage reliance on single metric optimisation. Here we formulate the PSEC challenge (Performance, Scalability, Environment and Cost) to integrate broader sustainability thinking with precise technical solutions and so enable successful commercialisation of these advanced materials.

Advanced nanomaterials have a central role to play in the pursuit of sustainable development goals. Applications span sectors, including energy, medicine and environmental clean-up. However, despite an explosion in their discovery and synthesis, these materials are struggling to make it through to commercial production. Their development is hampered by costly, resource intensive and scale-sensitive processes. Herein, we highlight widespread early-stage reliance on single metric optimisation as a primary cause of development failure and, conversely, emphasise the importance of multi-criteria thinking within both research design and execution, and particularly through discovery and design stages. We formulate the PSEC challenge (i.e. Performance, Scalability, Environment and Cost) as a means to integrate broader sustainability thinking with precise technical solutions. We propose overt emphasis on a correspondingly expanded specification of critical material attributes to better direct and integrate research. We highlight the potential for the development of MCDA (multi-criteria decision aiding) tools and opportunities for generating, consolidating, and extensively exploiting good quality whole-system data. Our paper represents a community call-to-action so that nanomaterial discoveries can reach the markets and fulfil their sustainable development potential.

## The PSEC challenge

### Advanced nanomaterials

Advanced nanomaterials form an important class of new materials, with the potential to contribute to sustainable development goals. Applications span sectors, including energy, drug delivery and environmental clean-up. Associated industrial manufacturing offers corresponding economic opportunities. Their unique properties (e.g. high surface area, catalytic reactivity and optical response) offer the high performance desired in many applications (e.g. catalysis, adsorption, electronic and energy harvesting/storage)^1, 2^ and there has been an explosion in nanomaterials discovery and synthesis, leading to many demonstrated and potential applications^3^. However, there is also a continuing failure of sustainable scale-up^4^. Material development is hampered by costly, resource intensive and scale-sensitive processes. These barriers make commercial realisation impossible, which in turn limits the ultimate contribution to sustainable development^5^. The situation is well illustrated by a recent inventory, which documented >5000 consumer products that contain nanomaterials^6^ and many more non-commodity products such as industrial catalysts and separation media. However, these are dominated by a small number of low to medium-value nanomaterials (e.g. titania, zinc oxide and silica), not exactly boundary-pushing technologies, thus highlighting barriers to the translation of high-value nanomaterials^7^.

### Sustainable Scale-up

**Fig. 1:**
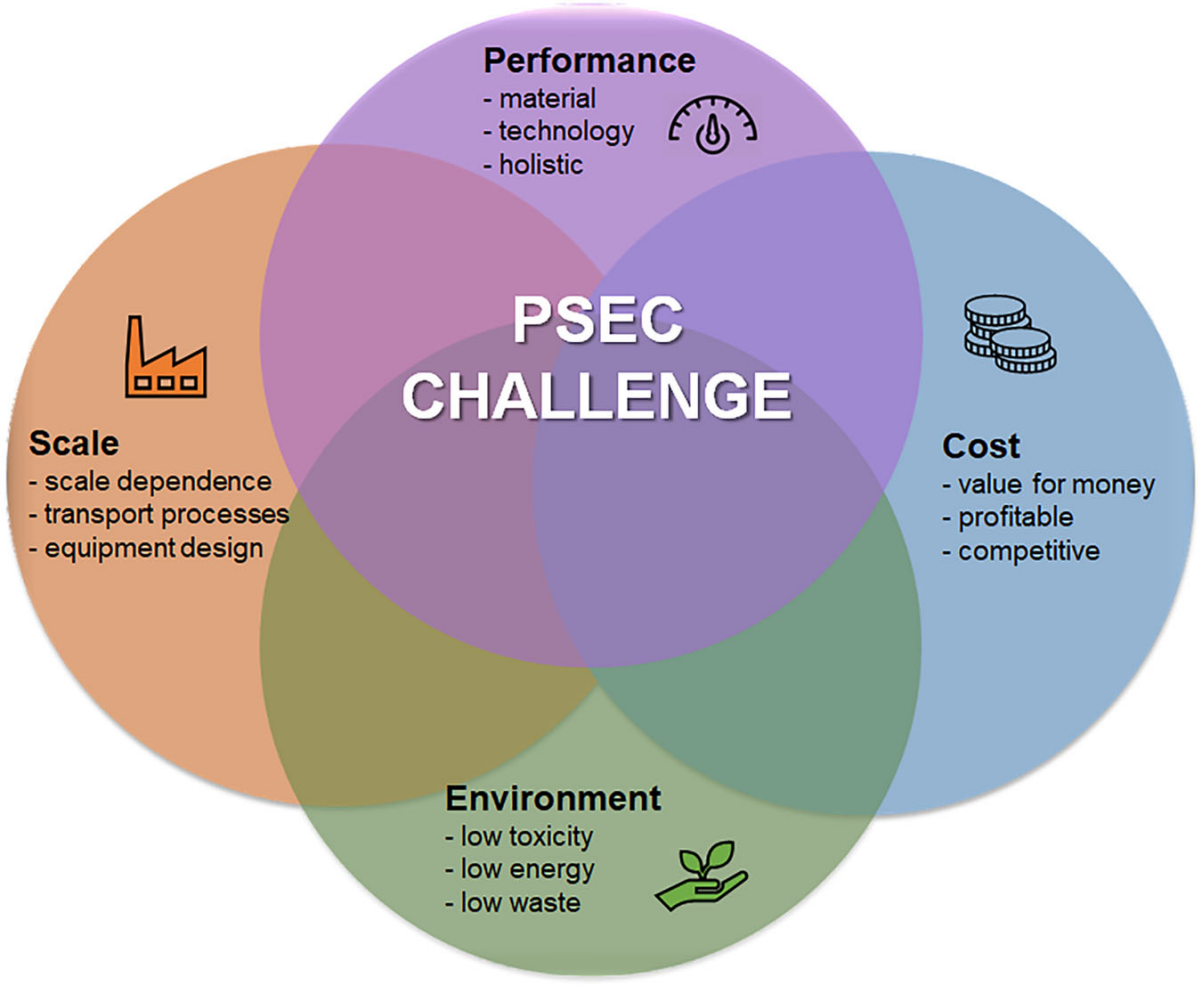
The PSEC development challenge for sustainable and scalable enabling technology. Thorough multi-criteria thinking can be achieved via the PSEC development challenge (i.e. Performance, Scalability, Environment and Cost) which will help address specific barriers to commercial success.

To realise the sustainable potential of advanced nanomaterials, their discovery, design and development need to minimise impacts and maximise benefits across the full remit of social, economic and environmental concerns. This is a diverse agenda and represents a complex multi-criteria challenge. Unfortunately, thorough multi-criteria thinking is rare in early-stage research. By contrast, our formulation of what we have called the PSEC development challenge (i.e. Performance, Scalability, Environment and Cost) pinpoints specific barriers to commercial success and emphasises the importance of multi-criteria thinking (Fig. 1).

Implications of the PSEC challenge, or rather of the systemic failure to recognise and address it, are far reaching, and present as four interconnected concerns:

- Poor scalability: Scaling high-value nanomaterials for industrial applications is complex and yet scalability is generally an afterthought. Established methodologies fail to escape the laboratory^7, 8^. The European Chemicals Agency recently highlighted that “the main barrier to market growth was reported to be in the scaling up of the manufacturing processes of all types of nanomaterials”^5^. The conditions and reagents employed render scale-up impractical, while scale-sensitivities lead to variable material quality and loss of performance. Studies may report that a synthesis is facile, cheap or readily scalable, but claims lack credibility if reactions are only performed at small scale (e.g. ≪1 g), rely on expensive and non-recyclable catalysts, reagents and solvents; give little considerations to downstream processing (e.g. separation or purification); or fail to consider the effects of transport phenomena (e.g. mixing, heat transfer)^9^.
- Unfavourable economics: The combination of intricate methods and the need for specialised reagents and equipment means that, even putting practical difficulties to one side, the intrinsic economics fail to add up. These new materials cannot compete with existing, low- or medium-value commercial products, and their cost is too high even to realise the potential of novel higher value applications. Metal organic framework (MOF) materials provide an example. MOFs exhibit very high surface areas (easily >1000 m^2^/g) with added features including selective adsorption and catalysis. However, typically, the literature focus is limited to MOF structure and surface area, while little attention is paid to economics, scalability or sustainability^10^. Technoeconomic analysis of MOF-5 synthesis has shown that due to solvent cost, degradation of solvents (leading to downstream purification issues) and low added value, they are less likely to be commercially viable without radically changing their synthesis^11^.
- Environmental Damage: Most methods for nanomaterials processing are energy and resource intensive^4,12^ (e.g. nanomaterials synthesis is found to be over 1000 times more wasteful compared to that of bulk chemicals^13,14^). Beyond the clear cost implications, these processes are also environmentally unsustainable. While considerable nanomaterial research claims to focus on green synthesis, these claims are rarely supported by convincing evidence or move beyond considering a single optimisation metric. Worryingly, the rhetoric and momentum of these studies can spread to misguided decisions both within and beyond the research community. A recent example includes the drive for replacing commercially used CdSe semiconductors because they “will not be a viable solution for real-life applications in the longer term, due to their (eco)-toxicity”^15^. This single metric view ignores the complete picture. Indeed, when the cumulative energy demand (CED) of CdSe was compared with less toxic InP alternatives, CdSe were found to use 10^2^ to 10^3^ times less energy^16^. CED is also a useful general predictor for other environmental impact categories^17^.
- Unmet Societal Needs: As described, nanomaterial development driven by a single or limited set of design criteria leads to development failure and the associated opportunity cost of unmet societal needs. The filters of public-perception and policy-making can add further challenges, potentially holding back otherwise game changing technical solutions or, indeed, promoting less technically desirable ones^4,7^. A multi-criteria design approach is better suited to engage with and respond to specific issues of perception and policy, and also to integrate this engagement with robust and balanced technical evaluation (e.g. identifying and responding to particularly influential data requirements or furnishing table-turning comparisons).

## The PSEC solution

There is an urgent need for improved research strategies and supporting methodologies, particularly through the discovery and design stage^18,19^. The scope and variety of implicated reaction systems, material properties, performance potential and application scope are enormous. The scale and diversity of corresponding research undertakings are similarly so. Instigating change within this landscape is a challenge, especially if it is to be achieved without compromising the flexibility and responsiveness of individual researchers and the idiosyncrasy of individual research missions.

Different approaches can support the desired evolution, ranging from engagement and networking, white papers, development of standards, shared methodologies and data consolidation, to the development of formal evaluation and decision-aiding tools. Ultimately, it is the aggregate quality of research design (and underlying decisions) that matters rather than, necessarily, whether a specific methodology or tool comes to dominate. At the same time, the scale of the challenge demands a concerted response. Consequently, tool development represents an attractive and coordinating objective.

In the case of complex tool-based development for practical and subjective decision environments, no matter how good the tool is technically, to make a difference, it must also find widespread acceptance and use. To this end community standards and methodology usefully precede and inform incremental development, making good use of principle-proofing and proto-typing.

It is also important to recognise that implicated decisions, even restricted to those through discovery and design stages are not universal and always depend on a particular purpose and context at a specific time. The design and evaluation of decision tools needs to be performed with relevant and specific use cases and use-persona in mind (who, what, why, when).

### Research design

Robust and transparent critical material attribute definition provides a pivotal reference for early-stage research and simultaneously provides a bridgehead for communication between upstream and downstream disciplines, not least in helping to define test methods and signal research needs. Consequently, overt emphasis on expanded ‘PSEC’ attribute-sets would provide stimulus for the development of tailored methods, which enable a balanced view of performance, scalability, techno-economics and environmental impacts.

Taken further, this logic erodes traditional delineations of upstream and downstream, replacing them with a more integrated mindset. Under this view, a systems approach can be employed to evaluate, prioritise and pursue multiple-interconnected research strands to drive pragmatic realisation of product development goals. This style has been elaborated and illustrated for bioinspired silica nanomaterials^20^. In practice this demands early-stage incorporation of:

- assessing feasibility of manufacturing, which requires measurement of time scales of reactions and transport processes such as heat transfer and mixing mechanisms, and understanding of associated dependence on production scale. For example, recently, the understanding of mixing during the synthesis of ZnO nanoparticles was utilised to scale-up their production^21^, while scale-independent mixing correlations were developed for green synthesis of silica nanomaterials, enabling rapid scale-up^22^.
- technoeconomic analysis, building a process flow diagram for industrial scale manufacturing and designing unit operations. This will help in identifying routes to manufacturing as well as highlighting potential process chemistry concerns. For example, problems associated with the downstream treatment of amine effluents from nanosilica production^23^, initiated fundamental research to develop a novel synthesis method, which enabled reuse of the amines, thereby avoiding the problem of discard^24^.
- early consideration of environmental/health implications. Full blown life cycle analysis may be a step too far but life cycle thinking, combined with the increasing array of green metrics and screening methodologies, can provide an effective and pragmatic approach. For example, recently, a simple-to-use metric was developed to quantify each of the 12 principles of green chemistry using readily available data^25^.

In addition to shared methodology, there is also the attractive proposition of establishing dedicated testbeds for rapid evaluation of candidate nanomaterials. These could, for example, offer the use of specialised reactors with a small footprint (e.g. impinging-jet, vortex or annular reactors) yet with the ability to map a wide range of flow and mixing conditions covering lab-to-plant scales, which forms an excellent avenue for experimentally evaluating scalability^21,26,27^.

### Critical research data

Irrespective of approach, the availability of good quality decision critical information provides a vital foundation. This in turn relies on the effective selection, collection, and consolidation of necessary data—both experimental and computational. Importantly, the expanded PSEC critical attributes of the previous section, demands correspondingly expanded critical data sets. Specific technical areas, which deserve greater attention include the following:

- Quantitative knowledge of reaction kinetics, which are critical in the design of a manufacturing process^28^.
- Experimental and simulation data to build models (empirical or from first principles) that describe synthesis-structure-property-performance relationships^29^.
- Equally important is the exploration of the fundamental basis of synthesis and activity through fundamental and mechanistic analysis.
- Process chemistry provides another important target, which demands particular attention to yield and conversions, and dependence of the synthesis on feedstock, solvents, and conditions. These data are extremely important for designing unit operations and manufacturing processes.

This shopping list has the potential to overwhelm the upstream researcher. Again, there is potential to mediate this challenge through the bridgehead offered by explicit and robust definition of critical material attributes, supported by accompanying standards and guidance. Even so, generating far reaching data remains a practical challenge given the number of experiments and simulations potentially required. Usefully, novel statistical and machine learning methodologies provide tractable means to map large reaction spaces^21^.

### Multi-criteria assessment

A comprehensive solution to the challenges described so far is offered by our concept of an integrated modelling-experimental methodology tailored for the development of scalable and sustainable advanced nanomaterials. This methodology would enable simultaneous search, evaluation and optimisation against multiple-criteria, not least performance, scalability, environment/health and cost.

Leading researchers in green chemistry have called for a framework to perform holistic multi-criteria assessments and inform design of emerging nano-products^29^. The European Commission’s Joint Research Centre also recently concluded that the absence of a holistic and systematic approach to assess sustainability at the material design stage^30^ is a crucial barrier in realising impact from nanomaterials. Multi-criteria decision aiding (MCDA) frameworks are highly suitable for the purpose, especially when interfaced with experimental campaigns since they need to improve iteratively through step-wise incorporation of targeted multi-criteria data. The end goal is robust and reliable predictive models able to identify and prioritise synthesis methods most likely to yield the targeted scalable and sustainable materials.

Survey work, performed by us and others of major sustainability assessment tools^4,13,14,31^ found that MCDA is the most relevant for conducting a sustainability assessment, while paying particular attention to the PSEC components – performance, scale-up, environment/health and cost. Approaches like this are required because, although various relevant evaluation tools exist, and are applied in the field of functional and high-value nanomaterials, none integrate the full-scope described above. Many existing tools such as life cycle assessment, whilst going into detail on the potential environmental impacts, do not look at the techno-economic feasibility or the societal need for a particular system^31^. These tools are often used in isolation and do not allow for informed decisions to be made in terms of selection of a specific manufacturing route or choice of feedstock material, and hence lack practical usefulness for advancing to higher technology readiness levels (e.g. scale-up and commercialisation)^13,14^.

MCDA methodologies provide a realistic way to combine multiple (categorically distinct) criteria^31,32^. Also that they are both flexible and specific, being tailorable to a target scenario, application or market need. Similar approaches are emerging in many industrial fields such as water and energy^33,34^ provides assurance of the principle. However, existing tools from other areas cannot be applied directly to nanomaterials because model function and reliability requires tuning to domain-specific needs and datasets. Equally, current applications within the area of sustainable nanomaterials are limited to small-scale chemical reactions, rather than to scalable and economically viable routes. Thus, further work is needed both in the development of sector-specific models and to secure underpinning data.

## Outlook

It is clear that change is needed so that nanomaterial discoveries can reach the markets and fulfil their sustainable development potential. We present the ideas in this paper as a community call to action and an opportunity for a decisive shift in mind-sets and approach.

Our formulation of the PSEC challenge focuses requirements and pinpoints specific technical barriers. It highlights widespread early stage reliance on single metric optimisation as a primary cause of development failure and, conversely, emphasises the importance of multi-criteria thinking.

**Fig. 2:**
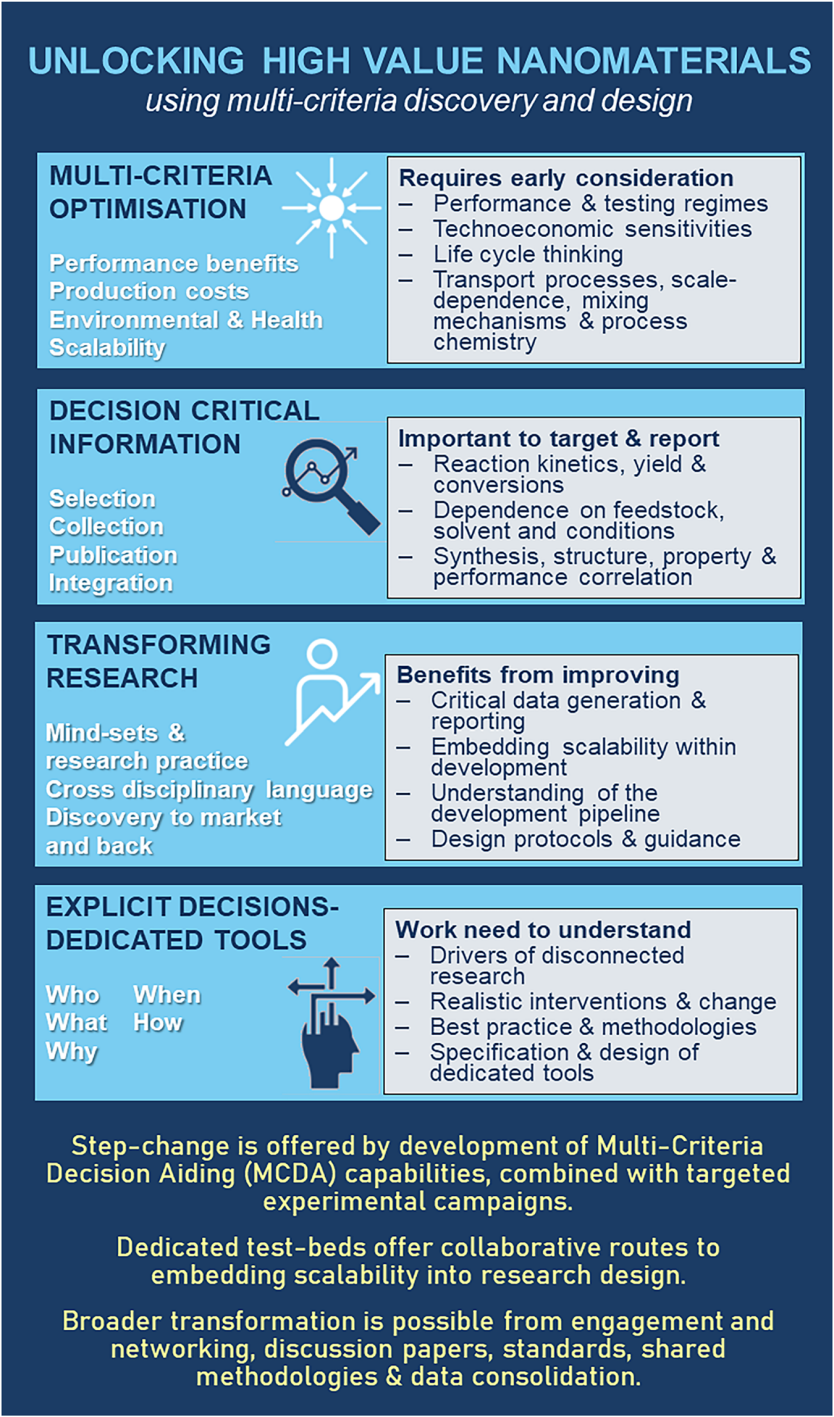
Avenues for addressing the PSEC challenge. A summary of key avenues for addressing PSEC challenges: embracing multi-criteria thinking, ensuring the availability of good quality decision-critical information, and making the best possible use of it within research design and execution.

There is an urgent need for improved research strategies and methodologies, especially through the early stages of research. These need to embrace multi-criteria thinking, ensure the availability of good quality decision critical information, and make the best possible use of it within research design and execution (Fig. 2).

Transforming research practices is challenging, especially if achieved without compromising the flexibility and responsiveness of individual researchers in pursuit of idiosyncratic research missions. Collaborating along traditional disciplinary lines is not enough, we need to be aware of complementary disciplines and develop common languages. These need to encompass the entire development pipeline and strengthen links between academia, industry, and the markets. Most importantly, thinking about scalability needs to be embedded throughout.
